# A 6-month supported online program for the treatment of persecutory delusions: Feeling Safer

**DOI:** 10.1017/S0033291725100676

**Published:** 2025-06-30

**Authors:** Daniel Freeman, Louise Isham, Jason Freeman, Laina Rosebrock, Thomas Kabir, Alex Kenny, Rowan Diamond, Ariane Beckley, Natalie Rouse, Memoona Ahmed, Felicity Hudson, Glory Sokunle, Felicity Waite

**Affiliations:** 1 Department of Experimental Psychology, University of Oxford, Oxford, UK; 2 Oxford Health NHS Foundation Trust, Oxford, UK; 3 Department of Psychiatry, University of Oxford, Oxford, UK; 4 The McPin Foundation, London, UK

**Keywords:** cognitive therapy, digital intervention, persecutory delusions, psychosis, schizophrenia

## Abstract

**Background:**

Based on an efficacious face-to-face theory-driven psychological therapy for persecutory delusions in the context of psychosis, we set out to develop a scalable guided 6-month online program. The aim was an intervention that patients can easily access and use, produces large clinical effects, and can be supported by a range of mental health professionals in less contact time than face-to-face therapy. We report here the proof-of-concept testing. At least moderate-sized clinical effects were required to progress to a randomized controlled trial (RCT).

**Methods:**

In the 6-month Feeling Safer online program, a certified medical device, patients complete a brief assessment and then are provided with up to 10 modules that match their difficulties. Regular remote meetings with a mental health professional also take place. These may be supplemented by in-person visits. A pre- to post-treatment cohort trial was conducted with 14 patients with persistent persecutory delusions. The primary outcome was the Psychotic Symptoms Rating Scale (PSYRATS)-Delusions.

**Results:**

Satisfaction and usability ratings of the program were high. Very large reductions in persecutory delusions were observed (PSYRATS mean reduction = 7.1, 95% C.I. = 3.4, 10.8, *n* = 13, Cohen’s *d* = 3.0). There were large improvements in paranoia, anxiety, depression, agoraphobic distress, psychological wellbeing, meaningful activity, personal recovery, recovering quality of life, and moderate improvements in insomnia, agoraphobic avoidance, and quality of life.

**Conclusions:**

The clinical effects associated with Feeling Safer were very high, comparable to those seen in the evaluations of the face-to-face therapy, and enable progression to an RCT.

## Introduction

Persecutory delusions – inaccurate beliefs that others intend harm – are one of the most common difficulties for people diagnosed with psychosis (Collin, Rowse, Martinez, & Bentall, 2023; Lemonde et al., 2021; Pappa et al., 2025). The delusions are too often resistant to standard treatments. Compared to treatment as usual, the effect sizes for first-generation cognitive behavior therapy for psychosis on positive symptoms is small (Cohen’s *d* = 0.3) (Bighelli et al., 2018; Salahuddin et al., 2024). Compared to placebo, the effect sizes for antipsychotics on positive symptoms are moderate (Cohen’s *d* = 0.45) (Leucht et al., 2017), though this varies by antipsychotic (Dong et al., 2024). Half of the patients with non-affective psychosis in current contact with clinical services have severe paranoia (Freeman et al, 2019). We have developed a psychological intervention, called Feeling Safe, that produces very large reductions in persistent persecutory delusions (Freeman et al., 2021). Feeling Safe is delivered by clinical psychologists in an average of 20 hours of in-person individual sessions over 6 months. The challenge is to get this new treatment to all patients who would benefit. In this paper, we report on the first clinical testing of a potential solution: an online version supported by a mental health staff member (Feeling Safer). By providing the intervention in an accessible version that can be used across mental health services, we aim to deliver significant improvements in outcomes for patients experiencing persecutory delusions.

Elsewhere, we have described the design principles underpinning Feeling Safe, including targeting key mechanisms, allowing for complexity, framing positive counterweights, building in measurement, and developing credibility and optimism (Freeman, 2024). These are perceptible characteristics of the intervention when it is used. Our empirically established theoretical model was used to identify the mechanistic targets (Freeman, 2016; Freeman, Isham, & Waite, 2025). Broadly, we consider the causation of persecutory delusions to be multi-factorial, meaning that intervention must target multiple factors. Causation will also vary to a degree across individuals, so any intervention must be personalized. We conceptualize persecutory delusions as inaccurate threat beliefs developed in the context of genetic and environmental risk. These beliefs are caused and maintained by a number of psychological processes, including excessive worry, low self-confidence, poor sleep, anomalous experiences, such as hearing voices, and safety-seeking behaviors. The clinical implication of the model is that safety must be relearned after having systematically weakened the influence of the maintenance factors.

Feeling Safe was largely built on a series of studies evaluating individual modular elements. These modules targeted specific maintenance factors. For example, in a randomized controlled clinical trial with 150 patients with persecutory delusions, we showed that our six-session worry intervention delivered over 6 weeks led to reductions in both levels of worry (Cohen’s *d* = 0.5) and delusions (Cohen’s *d* = 0.5) that were maintained at 6-month follow-up (Freeman et al., 2015). A mediation analysis showed that the changes in worry explained the changes in delusions. We also conducted a survey with 1800 patients with non-affective psychosis that assessed the prevalence of the maintenance factors identified in the model (e.g. worry, low self-confidence, insomnia) and patient treatment preferences (Freeman et al, 2019). It was found that the maintenance factors were very common in patients with severe levels of paranoia and that patients wanted help to reduce them. Hence the factors are sensible treatment targets. For example, it was found that 79% of patients with severe paranoia had clinically significant levels of worry and that when worry was present in patients with severe paranoia that 82% wanted help to worry less.

Modules targeting worry, self-confidence, the influence of voices, sleep, and re-learning safety while dropping safety-seeking behaviors were then combined into a full treatment. The 6-month program includes personalization of the treatment based on an initial assessment; patient preference in the modules to be tried; and session-by-session outcome measurement. We conducted an initial proof-of-concept test with 12 patients with persistent persecutory delusions in the context of non-affective psychosis (Freeman et al., 2016). The effect size improvement in delusion severity from pre- to post-treatment, as assessed by the Psychotic Symptoms Rating Scale (PSYRATS) – Delusions (Haddock, McCarron, Tarrier, & Faragher, 1999), was very large (Cohen’s *d* = 2.3).

This proof-of-concept testing led to a randomized controlled trial with 130 patients with persistent persecutory delusions in the context of non-affective psychosis (Freeman et al., 2021). The Feeling Safe program was compared to an alternative psychological approach (befriending) provided by the same therapists over the same time period. In this way, we could tell if the Feeling Safe program brings benefits beyond those that come with a positive therapeutic relationship. In the trial, patients received on average 20 hours of direct therapist contact time over 19 sessions, with an average of six and a half sessions spent outside for behavioral tests. Of the remaining sessions, approximately half were home visits and half were appointments in mental health clinics. There were no remote sessions. The Feeling Safe program led to large reductions in persecutory delusions, even above the alternative psychological therapy. At end of treatment, the effect size of Feeling Safe for overall delusion severity above the alternative therapy was large (Cohen’s *d* = 1.2), and this remained large at a 12-month follow-up (Cohen’s *d* = 0.9). The pre- to post-treatment change in persecutory delusions in the Feeling Safe group was very large (Cohen’s *d* = 2.8).

Provision of Feeling Safe by cognitive-behavioral therapists in face-to-face sessions, supported by 30 booklets written for the modules, is a mode of delivery suitable for many patients. However, its reliance on the availability and time of trained therapists limits both scalability and the amount of therapeutic content that can be covered. For the most effective theory-driven psychological therapies for anxiety disorders, guided online programs have proved an effective method to maintain high effect sizes and considerably reduce therapist time (Clark et al., 2023; Ehlers et al., 2023). Our goal was to develop a guided online version of Feeling Safe that patients could access when they wanted, that was easily used and appealing, and that could address a greater number of psychological processes (i.e. include an expanded range of therapeutic materials). We wanted a program that could: be supported by a range of staff groups; largely be delivered remotely to minimize the considerable time often spent travelling to patient homes; lessen the overall therapist time; and reduce the likelihood of therapist drift during intervention delivery. In general, guided or blended online interventions will see better treatment uptake and therefore clinical effects than self-guided (Buelens et al., 2023; Fairburn & Patel, 2017; Nunes-Zlotkowski et al., 2024). To reflect the variability in the patient group, we allowed flexibility in the level and type of support provided by the mental health staff member. Though the program was principally to be guided via brief weekly remote meetings between patient and mental health staff member, a limited number of in-person meetings could be held (i.e. we envisaged an optional degree of blended treatment).

Before proceeding to a planned randomized controlled trial (RCT) of Feeling Safer, we wanted evidence that it was easily usable, that patients were satisfied, and that it may be clinically efficacious. An *a priori* decision point for progression to the RCT was agreed with the funder: in proof-of-concept testing the intervention should be associated with at least a moderate effect size reduction (Cohen’s *d* = 0.6) in persistent persecutory delusions. The registered RCT (ISRCTN93974770) is a four-arm trial that tests delivery of Feeling Safer by each of three different staff groups (CBT therapists, assistant psychologists, and peer support workers) against treatment as usual (Freeman et al., 2025). We aim to gather evidence that a wide range of professionals can successfully deliver Feeling Safer. If the program is helpful, this should be evident when trained cognitive behavioral therapists support its delivery. Therefore, this is the staff delivery reference group. It was this group that administered the intervention in the proof-of-concept testing. The RCT is powered to determine whether the intervention works when supported by each of the three staff groups.

The primary hypothesis specified in the trial protocol of the proof-of-concept testing was that Feeling Safer will be associated with a reduction in persecutory delusions. The secondary hypotheses were that Feeling Safer will have high usability and satisfaction ratings and be associated with improvement in other psychiatric symptoms (depression, anxiety, insomnia, agoraphobia, and paranoia), psychological wellbeing, personal recovery, meaningful activity, and quality of life. We also planned to record adverse events and check medical notes for the occurrence of serious adverse events.

## Methods

### Design

The trial used a pre–post-cohort design. Participants were assessed before and after receiving Feeling Safer. The trial was run from a single center, with two NHS mental health trusts participating (Oxford Health NHS Foundation Trust; Berkshire Healthcare NHS Foundation Trust). Assessments were conducted by research assistants. Written informed consent was received from all participants. The trial was approved by the NHS Health Research Authority London – Harrow Research Ethics Committee (ref 23/LO/0951).

### Participants

The inclusion criteria for participants were willing and able to give informed consent for participation in the trial; aged 16 years or older; attending NHS mental health services for the treatment of psychosis; a persistent (at least 3 months) persecutory delusion (as defined by Freeman & Garety, 2000), held with at least 50% conviction; and no planned significant medication changes at the outset of participation. The exclusion criteria were a primary diagnosis of another mental health condition (e.g. substance use disorder) that would be the first clinical priority to treat; current engagement in any other intensive individual psychological therapy or a significant change in medication; in forensic settings or psychiatric intensive care unit; command of spoken English inadequate for engaging in the therapy; and significant learning difficulties that would prevent the completion of assessments or the therapy.

Thirty-two patients undertook an eligibility check. Fifteen people were ineligible, and three people were eligible but declined to participate. The reasons for ineligibility were not experiencing a persecutory delusion (*n* = 9), command of English not strong enough to complete the assessment (*n* = 1), unable to give informed consent (*n* = 1), and other (*n* = 4). The first patient began the study in April 2024, and the last assessment was conducted at the beginning of February 2025.

### The intervention

Feeling Safer is a guided online progressive web app recommended for adults (16 years or older) attending psychosis services who have a persecutory delusion. The software is intended to reduce persecutory delusions. Feeling Safer was designed and developed over 12 months, using the same principles as Feeling Safe (Freeman, 2024). Within the parameters of our brief to adapt Feeling Safe into a guided online program, we followed an iterative user-centered double diamond design process (Discover, Define, Develop, Deliver) (Design Council, 2005), involving people with lived experience, clinical psychologists, staff groups, designers, software developers, and a writer. The clinical lead developer for Feeling Safer was DF, with support in content development for some modules from LI and FW. The written text in the program was produced by JF. TK and AK led the patient involvement work. The user interface design and information architecture were created by a design studio, with feedback from user testing sessions.

Feeling Safer is a UKCA-marked, Class I medical device (standalone software as a medical device). It is available on all screen sizes, from desktops to mobile phones. Feeling Safer is standalone and not connected to NHS records. Personal information, such as full name, date of birth, or hospital number, is not entered into the program (individuals are simply sent a link by the health professional in order to log in). Patient personal data stored by the system comprise email address; mobile phone number (optional); progress through the modules, including assessments; messages between the patient and their mental health professional; and any notes in the patient’s private diary.

The treatment was programmed by a company that is ISO27001 and Cyber Essentials Plus accredited. (IS027001 is an international standard for the management of information security; Cyber Essentials is a UK government-backed set of standards for online security.) Both the design and development work to build the application were guided by AA Web accessibility standards, following industry best practice. Feeling Safer has been designed to meet the requirements of the Organization for the Review of Care and Health Apps (ORCHA). ORCHA provides independent assessment of health and medical apps to ensure compliance with current standards, regulation, and good practice and thus guarantee safety, security, and reliability.

Information is stored on an Azure-hosted database, which is encrypted at rest by default. (The user’s private diary is encrypted and stored on their device only.) Full database backups occur weekly; differential database backups occur every 12 hours. Regular checks are carried out for issues, such as software vulnerability, security incidents, and database backups. There is annual external penetration testing to detect vulnerabilities and security issues. Nightly automated checks include scans for vulnerable NuGet and NPM packages for management of code. Firewall logs and system logs are monitored weekly for evidence of suspicious activity. Every 3 months there is internal penetration testing of the system; testing of the database backups; manual inspection of the software to identify potentially vulnerable components; and review of the cryptographic art to identify if any cyphers have become vulnerable. Data in transit are protected by the use of Transport Layer Security 1.2 or higher (the recommended cryptographic protocol for protection of internet communications). The system uses Azure Active Directory (AD) authentication to prevent unauthorized access. Access requires two-factor authentication. A security breach process is in place.

The program is separated into three components that have separate portals for patients, deliverers, and system administrators. The Feeling Safer application allows patients to complete an assessment and then work through up to 10 digitized Feeling Safer modules. A mental health professional portal allows staff to view information about their patients’ use of the program (e.g. sessions completed, questionnaire scores). Finally, an administrator portal allows administrators to manage the professionals that may access the site. The application requires existing users to be logged in before they can interact with most of its functionality, leaving only selected pages to be accessed publicly (such as the login, privacy, and instructions for use pages). Only authorized staff can create a new user.

We wanted the patient experience to follow the logic of the original Feeling Safe intervention. They begin with an introductory module, which provides information about the therapy together with animations featuring patients recounting their personal stories. Patients then complete an assessment of the presence of potential maintenance factors in order for the program to provide only relevant treatment modules. The intervention is thus personalized. The presentation of the modules includes general recommendations for order of completion. The patient then works through a module of their choosing; once complete, they go on to the next relevant module. Throughout there are regular assessments, with information on progress fed back graphically to the user. Module content is conveyed by text and (optionally) voice; animations; and videos. Each module has an introductory animation (voiced by DF) and then includes animations featuring patient voices. Calendar functionality enables patients to schedule, and be reminded of, offline homework tasks. A ‘bad day’ section provides guidance for when people do not feel up to tackling a module. And there is also a private diary section (with audio or written input) where patients can keep notes. It was expected for patients to log in two or three times a week.

All modules currently comprising Feeling Safe were included, with the content substantially rewritten: getting better sleep; winning against worry; boosting self-confidence; feeling safe with voices; and finding safety. Five new modules were added: dealing with bad memories; getting active; finding balance (emotional regulation); getting the fears out (emotional expression) (Hepworth, Startup, & Freeman, 2011); and connecting with other people. Each module is broken down into many 10–15-minute sessions. These sessions include tasks to complete offline. The underpinning therapeutic approach is cognitive-behavioral.

The study is supported by a Lived Experience Advisory Panel made up of 11 people with experience of psychosis who live in the sites due to take part in the RCT. We also set up a UK-wide Involvement Network for Feeling Safer. The network comprises 30 people with relevant lived experience and includes diversity in age, gender, ethnicity, and location. We also engaged with several community groups. To date, 67 people with lived or caring experience of psychosis have contributed just over 400 hours of input. At the start of the project, seven in-person meetings were held across the country to gain insights into the potential opportunities and challenges in developing a guided online program. Issues discussed included fears about technology, inclusion, and how the program could be made especially engaging. Thirty-nine people with lived experience carried out a line-by-line review of all module content. Feedback was provided in written form and in 10 group meetings. The inclusion of a ‘bad day’ section was recommended by the lived experience advisors and the content compiled from their suggestions. The group also advised on the importance of user customization. As a result, patients can select a color scheme for the program, choose an avatar of the guide, and decide whether or not to hear the guide reading out the text. There was also ad hoc consultation on elements of the program (e.g. how best to display within-program assessment data). Iterative user testing took place during development. A final usability testing session with the completed program was conducted with six lived experience advisors, with excellent ratings.

It is expected that the mental health staff member supporting Feeling Safer meets with the patient at the beginning to explain the program, provide the access link, and check that the person is able to log in. This meeting is also important for developing a therapeutic alliance. Regular check-ins or meetings, typically weekly, with the mental health staff member are expected. These are conducted remotely (e.g. via telephone or video call). A smaller number of in-person sessions may be provided. These should typically be used for the staff member to help the person return to everyday activities or for behavioral tests to learn safety. The level – and type – of staff support can be tailored to a patient’s need. The staff-supported provision of Feeling Safer takes place over 6 months. Patients can still access the program after this period but without staff support. If a patient does not have a suitable device to access Feeling Safer, this is provided for them. The staff member supporting Feeling Safer is expected to have weekly clinical supervision. In the cohort study, Feeling Safer was supported by clinical psychologists (DF (three patients), LI (three patients), FW (three patients), LR (three patients), and RD (two patients). Two assistant psychologists also provided support for parts of the work of the clinical psychologists for three patients.

### Assessments

The primary outcome was the severity of persecutory delusions assessed by the Psychotic Symptoms Rating Scale (PSYRATS) (Haddock, McCarron, Tarrier, & Faragher, 1999). We included obtaining a 0–100% (do not believe to absolutely certain) rating of degree of conviction in the delusional belief (as used in the original Feeling Safe trial). Secondary symptom measures assessed were depression (PHQ-9) (Kroenke, Spitzer, & Williams, 2001), anxiety (GAD-7) (Spitzer, Kroenke, Williams, & Löwe, 2006), insomnia (Insomnia Severity Index) (Bastien, Vallieres, & Morin, 2001), agoraphobia (Oxford Agoraphobic Avoidance Scale) (Lambe et al., 2021), and paranoia (Revised Green et al Paranoid Thoughts Scale) (Freeman et al., 2021). Higher scores on these symptom scales all indicated greater severity. Also assessed were psychological well-being (Warwick-Edinburgh Mental Well-being Scale) (Tennant et al., 2007), personal recovery (Process of Recovery Questionnaire) (Law, Neil, Dunn, & Morrison, 2014; Neil et al., 2009), meaningful activity (time budget) (Jolley et al., 2006), and quality of life (EQ-5D-L and ReQol) (Herdman et al., 2011; Keetharuth et al., 2018). Higher scores on these measures indicated better wellbeing, quality of life, or functioning. Program usability was assessed with the Mobile App Rating Scale – adapted user version (Stoyanov et al., 2015) and therapy satisfaction with a Modified Client Satisfaction Questionnaire (Attkisson & Zwick, 1982).

Following completion of the final assessment, each participant’s medical notes were systematically checked for serious adverse events. The following adverse events were pre-specified as serious: death; suicide attempts requiring medical treatment; serious violent incidents; admission to hospital.

### Analysis

Paired t-tests were used to estimate mean differences and 95% confidence intervals for the pre–post assessments. The analysis did not include reporting p values, following the recommendation that ‘The analysis of a pilot study should be mainly descriptive or should focus on confidence interval estimation’ (Lancaster, Dodd, & Williamson, 2004). Effect sizes (Cohen’s *d*) were pre-specified to be calculated by dividing the change score obtained from the t-test by the standard deviation of the baseline average score. Presentation of satisfaction and usability data was descriptive. All statistical testing was conducted using SPSS Version 30.0 (IBM, 2024).

## Results

### Basic socio-demographic and clinical information

Information about the participants is summarized in Table 1. Levels of paranoia, categorized using the R-GPTS Part B, were average (*n* = 0), elevated (*n* = 0), moderately severe (*n* = 1), severe (*n* = 3), and very severe (10). The mean R-GPTS Part B score of 31.3 (SD = 7.6) was a little higher than the scores of the patients in the original Feeling Safe trial (mean = 27.1, SD = 8.4, *n* = 129). Levels of depression, categorized using the PHQ-9, were no depressive symptoms (*n* = 1), mild depressive symptoms (*n* = 0), moderate depressive symptoms (*n* = 1), moderately severe depressive symptoms (*n* = 8), and severe depressive symptoms (*n* = 4). Levels of anxiety, categorized using the GAD-7, were minimal (*n* = 0), mild (*n* = 1), moderate (*n* = 3), and severe (*n* = 10). The mean score on the Warwick-Edinburgh Mental Wellbeing Scale was 33.6 (SD = 8.4). This is indicative of low levels of psychological wellbeing and is comparable to the scores of patients in the original Feeling Safe trial (mean = 34.5, SD = 8.5, N = 130).

**Table 1.** Socio-demographic and clinical information

Variable	
Mean age in years (SD)	33.2 (9.4)
Gender (*n*) Male Female Other	9 4 1
Ethnicities (*n*) White White and Black Caribbean White and Asian Pakistani Black African	9 1 1 2 1
Working status (*n*) Unemployed Student Part-time employment Full-time employment	8 1 3 2
Marital status (*n*) Single Married/civil partnership	12 2
Outpatients (*n*)	14
Referral (*n*) Early intervention for psychosis Community mental health teams Psychological therapies pathway	5 7 2
Diagnosis (*n*) Schizophrenia Delusional disorder Schizo-affective diagnosis Psychosis NOS Depression with psychotic features Borderline personality disorder, with psychosis treatment occurring	2 2 4 4 1 1
Prescribed antipsychotic medication (*n*)	13
Prescribed antidepressant medication (*n*)	7

### Intervention uptake, satisfaction, and usability

All patients logged onto the program and completed the welcome module, including the assessment. After the assessment section, the average number of modules that were available to participants was 8.8 (SD = 1.0) (median = 9). The average number of modules undertaken by participants was 3.2 (SD = 2.1) (median = 3). The most common modules completed were boosting self-confidence (*n* = 11), winning against worry (*n* = 8), and finding safety (*n* = 7). Across the cohort all 10 modules were used at least once.

The average number of sessions (remote or in person) with the deliverer (or psychology assistant) was 21.1 (SD = 11.6), with an average total duration of 13.2 hours (SD = 7.1). The average number of in-person sessions was 4.0 (SD = 3.9), with an average total duration of 4.0 hours (SD = 3.9). For these in-person sessions, an average of 2.4 (SD = 3.0) sessions were spent outside for behavioral tests, with an average total duration of 2.5 hours (SD = 3.1). For the other in-person sessions, an average of 1.0 (SD = 1.2) of these sessions was held in the clinic and 0.6 (SD = 1.2) at home. The average number of remote sessions was 17.1 (SD = 11.0), with an average total duration of 9.2 hours (SD = 6.3). The mean total contact time in hours varied by therapist: 11.4 (SD = 5.7), 19.0 (SD = 2.1), 20.9 (SD = 0.5), 7.4 (SD = 4.9), and 9.8 (SD = 9.1).

Therapy satisfaction ratings are shown in Table 2. Ratings were high, and all patients thought that the program had made them feel safer, with the only reservation for a number of people being that they would have liked Feeling Safer for a longer period of time.

**Table 2.** Satisfaction with the Feeling Safer program

How would you rate the quality of the Feeling Safer program you have received? (*n*)				
	Excellent	Good	Fair	Poor
	9	4	0	0
To what extent has Feeling Safer helped you feel safer? (*n*)				
	Yes, it helped a great deal	Yes, it helped a bit	No, it did not really help	No, it seemed to make things worse
	8	5	0	0
What did you think of the length of the Feeling Safer program? (*n*)				
		Too short	Just the right length	Too long
		6	5	2
Was the member of staff supporting you with the Feeling Safer program helpful? (*n*)				
	Yes, they were very helpful	Yes, they were somewhat helpful	No, they did not really help.	No, they were very unhelpful.
	12	1	0	0
How likely are you to recommend Feeling Safer to friends and family if they needed similar care or treatment? (*n*)				
Extremely likely	Likely	Neither likely or unlikely	Unlikely	Extremely unlikely
7	6	0	0	0
In an overall, general sense, how satisfied are you with the Feeling Safer program you have received? (*n*)				
	Very satisfied	Mostly satisfied	Indifferent or mildly dissatisfied	Quite dissatisfied
	6	7	0	0

Usability ratings for the online program are shown in Table 3. In general, the online program was viewed as being of high quality in terms of functionality, aesthetics, and information. There is scope for improvement in how much users can customize the program and the level of interactivity. The overall program star ratings from the participants were one star (one of the worst apps I’ve used) (*n* = 0), two star (*n* = 0), three star (average) (*n* = 1), four star (*n* = 5), and five star (one of the best apps I’ve used) (*n* = 6). In terms of recommending Feeling Safer to people who might benefit from it, the ratings were: I would not recommend this app to anyone (*n* = 0), very few people (*n* = 1), several people (*n* = 1), many people (*n* = 2), and I would recommend this app to everyone (*n* = 8).

**Table 3.** Usability ratings for the Feeling Safer program (Mobile Application Rating Scale: user version)

Engagement				
Is the app interesting to use? Does it present its information in an interesting way compared to other similar apps? (*n*)				
Not interesting at all	Mostly uninteresting	OK, neither interesting nor uninteresting; would engage user for a brief time (< 5 minutes)	Moderately interesting; would engage user for some time (5–10 minutes total)	Very interesting, would engage user in repeat use
1	0	1	3	7
Does it allow you to customize the settings and preferences that you would like to (e.g. sound, content and notifications)? (*n*)				
Does not allow any customization or requires setting to be input every time	Allows little customization and that limits app’s functions	Basic customization to function adequately	Allows numerous options for customization	Allows complete tailoring to the user’s characteristics/preferences, remembers all settings
0	2	5	5	0
Does it allow user input, provide feedback, contain prompts (reminders, sharing options, notifications, etc.)? (*n*)				
No interactive features and/or no response to user input	Some, but not enough interactive features which limits app’s functions	Basic interactive features to function adequately	Offers a variety of interactive features, feedback and user input options	Very high level of responsiveness through interactive features, feedback and user input options
1	0	3	7	1
Is the app content (visuals, language, design) appropriate for the target audience? (*n*)				
Completely inappropriate, unclear or confusing	Mostly inappropriate, unclear or confusing	Acceptable but not specifically designed for the target audience. May be inappropriate/ unclear/confusing at times	Designed for the target audience, with minor issues	Designed specifically for the target audience, no issues found
0	0	1	1	10
Functionality				
How accurately/fast do the app features (functions) and components (buttons/menus) work? (*n*)				
App is broken; no/insufficient/inaccurate response (e.g. crashes/bugs/broken features, etc.)	Some functions work, but lagging or contains major technical problems	App works overall. Some technical problems need fixing, or is slow at times	Mostly functional with minor/negligible problems	Perfect/timely response; no technical bugs found, or contains a ‘loading time left’ indicator (if relevant)
0	0	0	7	5
How easy is it to learn how to use the app; how clear are the menu labels, icons and instructions? (*n*)				
No/limited instructions; menu labels, icons are confusing; complicated	Takes a lot of time or effort	Takes some time or effort	Easy to learn (or has clear instructions)	Able to use app immediately; intuitive; simple (no instructions needed)
0	0	2	2	8
Does moving between screens make sense; Does app have all necessary links between screens? (*n*)				
No logical connection between screens at all/navigation is difficult	Understandable after a lot of time/effort	Understandable after some time/effort	Easy to understand/navigate	Perfectly logical, easy, clear and intuitive screen flow throughout, and/or has shortcuts
0	0	0	5	7
Do taps/swipes/pinches/scrolls make sense? Are they consistent across all components/screens? (*n*)				
Completely inconsistent/confusing	Often inconsistent/confusing	OK with some inconsistencies/confusing elements	Mostly consistent/intuitive with negligible problems	Perfectly consistent and intuitive
0	0	1	5	6
Aesthetics				
Is arrangement and size of buttons, icons, menus and content on the screen appropriate? (*n*)				
Very bad design, cluttered, some options impossible to select, locate, see or read	Bad design, random, unclear, some options difficult to select/locate/see/read	Satisfactory, few problems with selecting/locating/seeing/reading items	Mostly clear, able to select/locate/see/read items	Professional, simple, clear, orderly, logically organised
0	0	0	4	8
How high is the quality/resolution of graphics used for buttons, icons, menus, and content? (*n*)				
Graphics appear amateur, very poor visual design – disproportionate, stylistically inconsistent	Low-quality/low-resolution graphics; low-quality visual design – disproportionate	Moderate-quality graphics and visual design (generally consistent in style)	High-quality/resolution graphics and visual design – mostly proportionate, consistent in style	Very high quality/resolution graphics and visual design – proportionate, consistent in style throughout
0	0	1	4	7
How good does the app look? (*n*)				
Ugly, unpleasant to look at, poorly designed, clashing, mismatched colors	Bad – poorly designed, bad use of color, visually boring	OK – average, neither pleasant, nor unpleasant	Pleasant – seamless graphics – consistent and professionally designed	Beautiful – very attractive, memorable, stands out; use of color enhances app features/menus
0	0	2	8	2
Information				
Is app content correct, well written, and relevant to the goal/topic of the app? (*n*)				
Irrelevant/inappropriate/incoherent/incorrect	Poor. Barely relevant/appropriate/coherent/may be incorrect	Moderately relevant/appropriate/coherent/and appears correct	Relevant/appropriate/coherent/correct	Highly relevant, appropriate, coherent, and correct
0	0	2	4	6
Is the information within the app comprehensive but concise? (*n*)				
Minimal or overwhelming	Insufficient or possibly overwhelming	OK but not comprehensive or concise	Offers a broad range of information, has some gaps or unnecessary detail; or has no links to more information and resources	Comprehensive and concise; contains links to more information and resources
0	0	1	4	7
Is visual explanation of concepts – through charts/graphs/images/videos, etc. – clear, logical, correct? (*n*)				
Completely unclear/confusing/wrong or necessary but missing	Mostly unclear/confusing/wrong	OK but often unclear/confusing/wrong	Mostly clear/logical/correct with negligible issues	Perfectly clear/logical/correct
0	0	0	3	8
Does the information within the app seem to come from a credible source? (*n*)				
Suspicious source	Lacks credibility	Not suspicious but legitimacy of source is unclear	Possibly comes from a legitimate source	Definitely comes from a legitimate/specialized source
0	0	1	1	9

The perceived impact of the online program is summarized in Table 4. Almost all patients reported increases in understanding, awareness of the importance of the issue, and intent to change.

**Table 4.** Perceived impact of Feeling Safer (Mobile Application Rating Scale: user version)

This app has increased my awareness of the importance of addressing ‘feeling safer’ (*n*)				
1 – Strongly disagree	2	3	4	5 – strongly agree
0	1	1	5	5
This app has increased my knowledge/understanding of ‘feeling safer’ (*n*)				
1 – Strongly disagree	2	3	4	5 – strongly agree
0	1	0	6	5
The app has changed my attitudes toward improving ‘feeling safer’ (*n*)				
1 – Strongly disagree	2	3	4	5 – strongly agree
0	1	1	4	6
The app has increased my intentions/motivation to address ‘feeling safer’ (*n*)				
1 – Strongly disagree	2	3	4	5 – strongly agree
0	1	0	3	8
This app would encourage me to seek further help to address ‘feeling safer’ (if I needed it) (*n*)				
1 – Strongly disagree	2	3	4	5 – strongly agree
0	1	0	5	6
Use of this app will increase ‘feeling safer’ (*n*)				
1 – Strongly disagree	2	3	4	5 – strongly agree
0	1	2	3	6

### Clinical outcomes

One participant did not provide outcome data after being discharged from mental health services. He had improved and then moved out of the area without leaving new contact details.

The outcome measure scores are summarized in Table 5. The overall change in persecutory delusions was very large (see Table 4). The actual change in PSYRATS scores is remarkably close to that seen in the RCT test of the face-to-face therapy (in which PSYRATS scores reduced from 18.5 (SD = 2.3) to 11.6 (SD = 5.9)). The percentage reductions in the PSYRATS total score were 86, 70, 67, 65, 48, 44, 39, 38, 19, 17, 5, 0, and -18. Less change in the delusion was (non-significantly) associated with a greater number of sessions with the therapist, *r* = 0.3, *p* = .311, and greater total time with the therapist, *r* = 0.5, *p* = .086.

**Table 5.** Outcome measure scores

Assessment	Baseline		End of intervention		Paired samples t-tests		
	n	Mean (SD)	n	Mean (SD)	Mean difference	95% confidence intervals	Effect size (Cohen’s *d*)
Primary outcome							
Persecutory delusion severity (PSYRATS)	14	18.6 (2.4)	13	11.5 (5.5)	7.1	3.4, 10.8	3.0
Secondary outcomes							
Conviction in the persecutory delusion (0–100%)	14	83.9 (13.0)	13	44.2 (36.0)	38.8	17.1, 60.5	3.0
Paranoia: reference (R-GPTS Part A)	14	19.4 (8.8)	12	10.6 (11.0)	8.3	1.5, 15.2	1.1
Paranoia: persecution (R-GPTS Part B)	14	31.3 (7.6)	12	16.3 (14.5)	13.8	4.3, 23.4	1.8
Depression (PHQ–9)	14	16.9 (4.9)	13	11.2 (7.1)	5.6	1.4, 9.8	1.1
Anxiety (GAD–7)	14	16.1 (4.3)	13	10.5 (6.4)	5.5	1.9, 9.1	1.3
Insomnia (ISI)	14	11.1 (8.0)	13	9.5 (8.6)	1.5	−2.7, 5.8	0.6
Agoraphobic avoidance (O-AS)	14	2.4 (2.5)	12	1.1 (2.1)	1.5	−0.07, 3.1	0.6
Agoraphobic distress (O-AS)	14	43.2 (24.4)	12	25.2 (23.6)	18.4	−0.9, 37.7	0.8
Psychological well-being (WEMWBS)	14	33.6 (8.4)	13	43.1 (10.3)	8.4	4.5, 12.3	1.0
Personal recovery (process of recovery questionnaire)	14	23.4 (11.7)	12	36.5 (14.3)	12.8	5.4, 20.1	1.1
Meaningful activity (time budget)	14	54.1 (13.6)	9	70.0 (16.1)	14.1	4.2, 24.0	1.0
Quality of life (EQ–5D-L index)	14	0.58 (0.27)	13	0.71 (0.28)	0.13	−0.04, 1.68	0.5
Quality of life (EQ–5D-L) – Health Today	14	38.7 (24.1)	13	54.6 (27.3)	13.1	2.4, 23.8	0.5
Quality of life (ReQol)	14	28.7 (16.2)	12	45.2 (18.0)	14.8	6.4, 23.1	0.9

All secondary outcomes showed improvements. The largest were seen for paranoia, anxiety, depression, personal recovery, meaningful activity, and psychological wellbeing (effect sizes of one or above).

### Adverse events

There was one serious adverse event (a violent incident) that was judged by the team and the trial’s Data Monitoring and Ethics Committee as unrelated to the treatment or trial procedures (the patient had a history of similar incidents). The same person also had a minor violent incident that was not serious (and not related to the trial).

Three other patients had adverse events that were not serious. One person had two instances of self-harm that did not require treatment. Another person had three A&E visits due to a physical health problem. Both patients had pre-existing histories of the occurrence of these events and none of the new events were judged related to the trial. One patient had a minor adverse event related to the intervention when the online program inadvertently displayed an error message that caused distress. This programming bug was then fixed.

There were no deaths, hospital admissions, suicide attempts, or complaints.

## Discussion

Before progressing to fuller clinical testing, it is crucial to thoroughly investigate a new treatment’s usability, engagement, and potential clinical value. As such, Phase I proof-of-concept testing is a vital stage. Here, we have looked in detail at Feeling Safer, the first extended guided online program for persecutory delusions. The patients who took part in the testing had severe and persistent persecutory delusions, accompanied by high levels of anxiety and depression. Their psychological wellbeing was very low. Several participants had expressed significant fears about technology beforehand. Yet uptake of the intervention was high, patients clearly liked it, and the program was considered highly usable. There is a straightforwardness to the design of the online program, developed in workshops and user testing sessions, that enables patients to navigate it easily. The content of the program was germane. After completing the assessment embedded in the program, each patient was presented with many modules and the content was judged as relevant and appropriate. All modules received use. The animations, voiced by patients, were especially powerful for participants. The patients thought that the program had helped them feel safer. The degree of change in persecutory delusions was extremely large. There were also substantial improvements in paranoia, anxiety, depression, psychological wellbeing, personal recovery, and meaningful activity. The supported online program may well match the benefits of the original face-to-face therapy. Proceeding to a RCT is clearly warranted.

We expected that patients might require different amounts, and types, of contact. The degree and nature of therapist support was therefore intentionally flexible. On average, direct deliverer contact time for Feeling Safer was two thirds of that for the original face-to-face version. The most substantial saving in therapist time, however, was due to far less travelling to appointments. There were clear differences between therapists in the total amount of contact time provided. In practice, and as the majority of therapists did, the intervention can be delivered in half of the original face-to-face contact time. There is clearly variability in how therapists may wish to support patients. Patients who were not responding tended to have the most contact time. These are the first patients to complete Feeling Safer. Three patterns were observed. First, a number of patients worked through several modules, a few sessions a week, and implemented changes in their lives based on the tasks suggested in the modules. The calls with the therapist were mainly used to reflect on progress and think about longer-term goals. Second, several patients proceeded quickly through all their allocated modules. Therapist contact was then used to revisit key modules methodically and plan implementation of the suggested techniques. Third, a small number of patients barely used the online program but did attend a number of remote sessions with the therapist. Many other patterns of use are likely to be seen when a greater number of people use the program.

There are a number of study limitations. The pre–post cohort design limits the level of confidence with which the clinical improvements can be ascribed to the intervention. Although the delusions were persistent, and had often been present for many years, it is likely that some of the change was simply due to time and the repetition of the assessments. The study design also does not allow us to determine which elements of the intervention may have had efficacy: perhaps the therapist contact rather than the online program led to improvements or vice versa. Feeling Safer was supported by expert therapists, which may have increased the size of effects. On the other hand, the fact that this was the very first delivery of the intervention may have reduced the size of effects: we are still learning how best to support Feeling Safer. There have also been additional features and functionality added to the online program. The demographic and clinical characteristics of the participants were similar to those reported in other clinical trials of interventions for persecutory delusions. But we do not know how representative they were of all patients in clinical services. This could have biased the results. The evaluation relied on quantitative testing. Adding qualitative interviews could have increased our depth of understanding. For this reason, our RCT will include an embedded qualitative study. An important limitation of the intervention is that not all patients benefit. A minority of patients will therefore require a different approach. Nevertheless, Feeling Safer is clearly feasible to deliver, acceptable to patients, and potentially highly efficacious.
